# An Unexpected Manifestation of Non-islet Cell Tumor Hypoglycemia in Pancreatic Ductal Adenocarcinoma

**DOI:** 10.7759/cureus.92384

**Published:** 2025-09-15

**Authors:** Talal Alomar, Jasmine Kaur, Mohamad Horani, Sammy Alomar, Iyad Syoufi

**Affiliations:** 1 Internal Medicine, University of California Los Angeles, Los Angeles, USA; 2 Internal Medicine, Chandler Regional Medical Center, Chandler, USA; 3 Internal Medicine, Chandler Regional Medical Center, Arizona, Chandler, USA; 4 Internal Medicine, Creighton University School of Medicine, Omaha, USA; 5 Endocrinology, Honor Health, Scottsdale, USA

**Keywords:** ductal pancreatic adenocarcinoma, igf-2 induced hypoglycemia, pancreatic cancer and hypoglycemia, paraneoplastic hypoglycemia, paraneoplastic syndrome

## Abstract

Pancreatic ductal adenocarcinoma (PDAC) is an aggressive malignancy accounting for more than 90% of pancreatic cancer cases and is rarely associated with paraneoplastic syndromes. Non-islet cell tumor hypoglycemia (NICTH), typically mediated by insulin-like growth factor 2 (IGF-2), is well-described in mesenchymal tumors and hepatocellular carcinoma but has not been documented in PDAC.

We report an 81-year-old man with stage IV PDAC who presented with profound hypoglycemia (glucose 32 mg/dL) in the absence of diabetes or hypoglycemic agents. Despite repeated dextrose infusions, his hypoglycemia persisted, necessitating intensive care unit (ICU) admission. Workup excluded insulinoma, autoimmune hypoglycemia, and adrenal insufficiency. An elevated IGF-2/IGF-1 ratio (457/7) was diagnostic for IGF-2-mediated hypoglycemia. The patient’s glucose stabilized with total parenteral nutrition, but he succumbed shortly thereafter to cancer-related complications. This is the first documented case of IGF-2-mediated NICTH in PDAC, underscoring the need to consider paraneoplastic hypoglycemia in atypical presentations of pancreatic cancer.

## Introduction

Pancreatic adenocarcinoma, primarily pancreatic ductal adenocarcinoma (PDAC), originates from the exocrine cells of the pancreas and represents the majority of pancreatic cancers. PDAC is infamous for its poor prognosis and aggressive biology, accounting for more than 90% of cases [1,2]. Clinical presentations typically include jaundice, abdominal pain, anorexia, and weight loss due to local invasion and bile duct obstruction [1,2].

Paraneoplastic syndromes are relatively rare in PDAC compared with pancreatic neuroendocrine tumors (PNETs), which more frequently secrete hormones such as insulin, glucagon, or gastrin [3,4]. NICTH is one such paraneoplastic phenomenon, characterized by recurrent hypoglycemia due to tumor-derived insulin-like growth factor 2 (IGF-2) production. Although extensively described in mesenchymal tumors, hepatocellular carcinoma, adrenal cortical carcinoma, and sarcomas [5-7], its association with PDAC has not previously been confirmed. Here we describe a case of PDAC complicated by severe, persistent hypoglycemia secondary to IGF-2 secretion. This case expands the known spectrum of paraneoplastic manifestations in PDAC and underscores the diagnostic challenges of unexplained hypoglycemia in cancer patients.

## Case presentation

An 81-year-old man with stage IV PDAC and liver metastases on chemotherapy presented to the emergency department with progressive fatigue. On arrival, he was ill-appearing but alert, with stable vital signs. Physical examination revealed abdominal distention without focal neurological deficits. Laboratory evaluation showed hemoglobin 9.2 g/dL, aspartate aminotransferase (AST) 62 U/L, alkaline phosphatase 463 U/L, and a critically low glucose of 32 mg/dL. There was no history of diabetes or use of insulin, sulfonylureas, or other hypoglycemic medications. Despite repeated intravenous dextrose 50% (D50W) boluses, his hypoglycemia recurred, prompting transfer to the intensive care unit. Endocrine evaluation was initiated to rule out common etiologies of hypoglycemia. Fasting insulin (<1 μIU/mL) and C-peptide (0.1 ng/mL) were suppressed, excluding insulinoma. Insulin autoantibody testing was negative, decreasing suspicion for autoimmune hypoglycemia. A normal cortisol stimulation test ruled out adrenal insufficiency.

**Figure 1:**
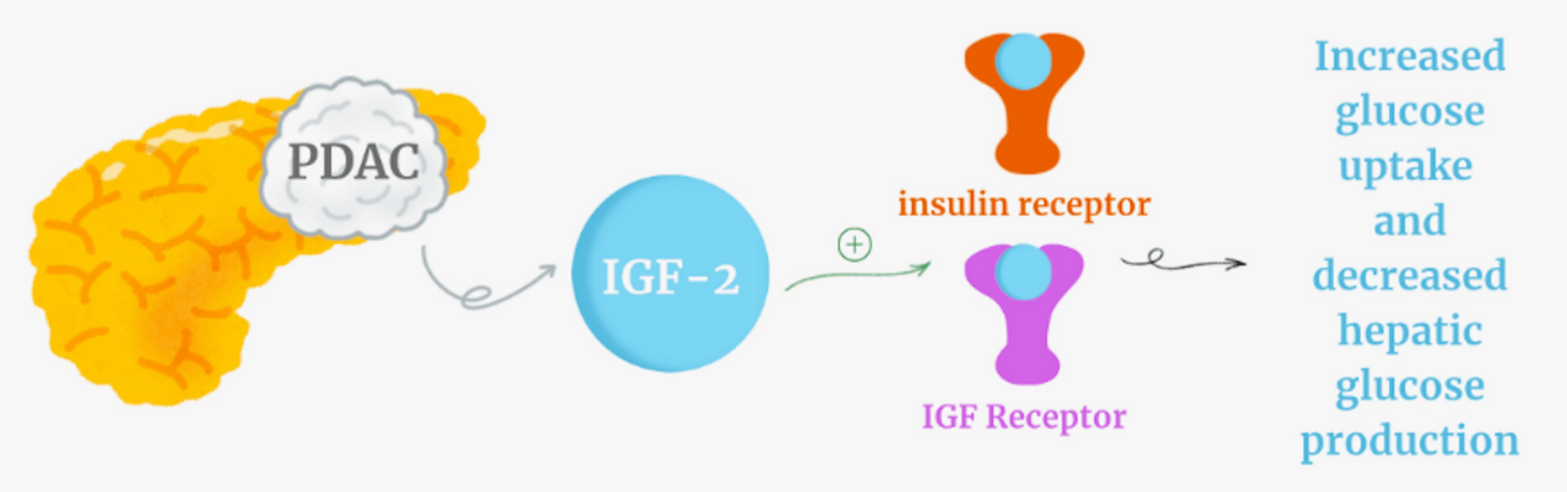
IGF-2 mediated hypoglycemia Diagram showing how insulin-like growth factor 2 (IGF-2) can activate both insulin receptors and IGF receptors to cause hypoglycemia. The image is created by the author Jasmine Kaur.

Given the known tumor burden, NICTH was suspected. Measurement of insulin-like growth factors revealed an IGF-2 level of 457 ng/mL (reference range: ~333-967 ng/mL) and an IGF-1 level of 7 ng/mL (reference range: ~50-300 ng/mL), confirming an elevated IGF-2/IGF-1 ratio diagnostic for IGF-2-mediated hypoglycemia [8] (Figure 1). The patient was initiated on total parenteral nutrition (TPN), resulting in stabilization of glucose levels. Unfortunately, his overall course remained poor, and he died shortly after hospitalization due to progression of his malignancy.

## Discussion

Pancreatic cancer encompasses a diverse group of malignancies arising from distinct cellular lineages within the pancreas. The most common type, pancreatic ductal adenocarcinoma (PDAC), originates from the exocrine ductal cells responsible for producing digestive enzymes. PDAC accounts for over 90% of pancreatic cancer cases and is characterized by its aggressive biology, often presenting with jaundice, abdominal pain, and weight loss due to bile duct obstruction [1,2]. While PDAC typically remains localized to the pancreas and surrounding structures, it rarely produces paraneoplastic conditions-systemic effects caused by tumor-secreted factors or immune responses [2]. Notably, rare paraneoplastic neurologic syndromes have been reported in PDAC, such as Lambert-Eaton myasthenic syndrome, underscoring that systemic effects, while uncommon, can occur [9].

In contrast, pancreatic neuroendocrine tumors (PNETs) arise from the endocrine islet cells and comprise only 5-10% of pancreatic cancers [3,4]. PNETs are frequently hormone-secreting and are categorized by the specific hormones they produce. For instance, insulinomas secrete insulin, causing recurrent hypoglycemia, while glucagonomas produce glucagon, leading to hyperglycemia and systemic catabolic effects. This distinction between PDAC and PNETs is critical: although both originate from the pancreas, they differ markedly in pathophysiology, prognosis, and treatment approaches. Whereas PNETs often declare themselves through hormonal syndromes, PDAC very rarely presents with systemic endocrine complications.

Insulin-like growth factors (IGF-1 and IGF-2) are peptide hormones with essential roles in growth, development, and cellular regulation. IGF-1 is primarily secreted by the liver under the stimulation of growth hormone (GH) and mediates many of GH’s anabolic effects, including tissue repair and proliferation [10,11]. IGF-2, also predominantly produced by the liver, is vital during fetal development, driving cellular proliferation and differentiation [12]. Dysregulation of the IGF system has been implicated in tumorigenesis, with evidence that cancers such as hepatocellular carcinoma and colorectal carcinoma may exploit overproduction of IGF-2 to sustain autocrine and paracrine signaling that enhances proliferation, invasion, and survival [13,14].

Hypoglycemia in NICTH results from tumor-driven IGF-2 excess. At high concentrations, IGF-2 can bind to insulin receptors, mimicking insulin activity. This leads to increased glucose uptake in peripheral tissues while simultaneously suppressing hepatic gluconeogenesis [11,14]. Furthermore, when IGF-2 production overwhelms the binding capacity of IGF-binding proteins (IGFBPs), the proportion of free IGF-2 rises, amplifying its hypoglycemic effects [12]. The biochemical signature is thus hypoglycemia with suppressed insulin and C-peptide, and an elevated IGF-2/IGF-1 ratio, precisely the findings in our patient.

While IGF-2-mediated hypoglycemia is well documented in association with mesenchymal tumors, hepatocellular carcinoma, gastrointestinal stromal tumors, and adrenal cortical carcinoma [5-7], confirmed cases in PDAC are virtually absent from the literature. Sharma et al. [15] and Diasio et al. [16] reported hypoglycemia in pancreatic carcinoma, but neither report demonstrated IGF-2 elevation as the mechanism. De Groot et al. reviewed 233 cases of NICTH, identifying fibrous tumors and hepatocellular carcinoma as the most common etiologies, with no cases attributable to PDAC [6]. Similarly, Yu et al. emphasized the rarity of IGF-2-mediated hypoglycemia outside a narrow range of tumor types [7]. Bodnar et al. later reviewed 288 cases of NICTH and recognized a spectrum of associated tumors, including sarcomas, renal cell carcinoma, and adrenal cortical carcinoma, but again, PDAC was not represented [5].

Our case, therefore, represents an extreme outlier in the literature-one of the first confirmed instances of IGF-2-mediated hypoglycemia in PDAC. This underscores that while NICTH is a recognized paraneoplastic syndrome, its occurrence in PDAC remains exceedingly rare. Recognition of this phenomenon is critical, as paraneoplastic hypoglycemia can be life-threatening and may confound clinical care in advanced cancer patients.

## Conclusions

This case illustrates a previously unreported association between PDAC and IGF-2-mediated hypoglycemia. It highlights the need for clinicians to consider paraneoplastic hypoglycemia in patients with unexplained hypoglycemia and solid organ malignancies, even those not classically associated with IGF-2 secretion. Recognition of this rare complication expands our understanding of PDAC’s systemic manifestations.
